# Dual-Purpose Vermicompost for the Growth Promotion and Suppression of Damping-Off Disease on Potted Vegetable Soybean

**DOI:** 10.3390/plants13121607

**Published:** 2024-06-11

**Authors:** Alongkorn Nonthapa, Chuleemas Boonthai Iwai, Sompong Chankaew, Shanerin Falab

**Affiliations:** 1 Department of Entomology and Plant Pathology, Faculty of Agriculture, Khon Kaen University, Khon Kaen 40002, Thailand; alongkornn@kkumail.com; 2 Department of Soil Science and Environment, Faculty of Agriculture, Khon Kaen University, Khon Kaen 40002, Thailand; 3 Integrated Land and Water Resource Management Research and Development Center in Northeast Thailand, Khon Kaen University, Khon Kaen 40002, Thailand; 4 Department of Agronomy, Faculty of Agriculture, Khon Kaen University, Khon Kaen 40002, Thailand; somchan@kku.ac.th; 5 Plant Breeding Research Center for Sustainable Agriculture, Khon Kaen University, Khon Kaen 40002, Thailand

**Keywords:** *Athelia rolfsii*, biocontrol, *Eisenia fetida*, *Eudrilus eugeniae*, soybean, sustainable agriculture

## Abstract

Vermicompost is applied as a soil amendment to promote plant growth and yield. It also helps to significantly reduce the incidence of soil-borne diseases. However, its efficiency depends on the type of earthworm from which it is formed. The current study aims to compare the effects of two vermicompost types derived from African nightcrawler (AF) and Tiger worm (TG) as a soil amendment to evaluate its potential for suppressing damping-off disease both in vitro and in vivo. It also aims to determine the effects of both vermicompost types on the growth and yield-related traits of potted Thai vegetable soybean [*Glycine max* (L.) Merrill] variety “Chiang Mai 84–2” grown under greenhouse conditions when amended to the soil at various application rates (1%, 2%, 3% *w*/*w*). AF vermicompost exhibited better suppression of damping-off disease than TG vermicompost in vitro and under greenhouse conditions. AF vermicompost performed significantly greater suppressive efficacy on the mycelial growth of *Athelia rolfsii* in vitro than TG vermicompost, indicated by 50% and 16% inhibition, respectively. Damping-off incidence on vegetable soybean seedlings grown in soil amended with AF vermicompost was significantly lower (21%) than in soil amended with TG vermicompost (32%) under greenhouse conditions. With an increased application rate of 1% to 2% or 3% for each vermicompost type, plant yields significantly enhanced, with no significant variations among the 2% and 3% rates. Applying vermicompost at 2% *w*/*w* through soil amendment was the appropriate rate for promoting the growth and yield of potted vegetable soybeans. The results can be used to guide the application of vermicompost to control soil-borne plant diseases, promote plant growth, and enhance yields, especially in terms of organic crop production. Further research is needed to evaluate its potential for other potted crops and protect against soil-borne diseases.

## 1. Introduction

Vermicompost is a nutrient-rich organic matter derived from the decomposition of bio-residues through earthworm digestion facilitated by gut-associated microorganism activity [1]. It is proven to promote plant growth as an organic fertilizer, being an alternative to chemical fertilizers [2,3] used in organic crop production [4]. Additionally, vermicompost has a broad range of benefits for plant production, including suppressing soil-borne disease, enhancing soil characteristics, and ameliorating abiotic stress [5,6]. The physico-chemical properties of vermicompost depend on the earthworm species when the same feeding materials are applied in the process [7]. Thus, depending on the earthworm species used in composting, it is necessary to investigate their varied responses to plant growth promotion and the suppression of disease.

Growing media amendment is an effective method of applying vermicompost to enhance plant growth and yield [8]. Plants grown in potting media amended with vermicompost in an optimal quantity demonstrate better performance than those grown in pure potting media [9,10]. Vermicompost produced by different earthworm species has been empirically demonstrated to promote plant growth and yield [11,12]. For example, mixing vermicompost derived from the African nightcrawler (*Eudrilus eugeniae* Kinberg, 1867), Asian blue worm (*Perionyx excavatus* Perrier, 1872), and Tiger worm (*Eisenia fetida* Savigny, 1826) into potting media of 50% by volume revealed significantly higher growth in chili than that grown in pure potting media [11]. Vermicompost from the African nightcrawler and Tiger worm is globally used in agriculture [12].

Importantly, the proportion of vermicompost for growth substrate amendment can be adjusted to an appropriate ratio according to crop demands and chemical substrate properties [13]. Several experiments have reported that soil amendment using varying proportions of vermicompost from the African nightcrawler [14,15,16] and Tiger worm [9,16,17,18] could determine the minimal requirements for the growth and yield promotion of specific plant species [4]. For the African nightcrawler vermicompost, soil amended with 15% (*w*/*w*) vermicompost led to the enhanced growth of potted tomato and eggplant [16], while 3% (*w*/*w*) vermicompost amendment to potting media resulted in greater tomato plant height, stem girth, and leaf numbers than plants grown in pure potting media [15]. Likewise, a significant promotion of growth and productivity on potted soybean, tomato, and eggplant was observed when plants were grown in potted media amendment using vermicompost at a rate of 148 kg N ha^−1^, compared to non-amended growing media [14]. In the case of Tiger worm vermicompost, tomatoes and eggplant grown in potted soil amended with 16% vermicompost produced higher yields than those in soil mixed with lower vermicompost percentages and non-amended soil [16]. Additionally, when incorporating Tiger worm vermicompost at 20% by volume into the potted soil, marigolds exhibited a higher percentage of germination and growth compared to plants grown in soil alone [18]. Maji et al. [17] also reported that mixing Tiger worm vermicompost with 9 g kg^−1^ of soil boosted growth, root nodulation, and yield in potted peas.

On the other hand, vermicompost as a soil amendment exhibits the potential to control soil-borne fungal plant diseases [19,20]. For example, soil amendment with vermicompost from the African nightcrawler at 20% (*w*/*w*) demonstrated suppression of damping-off disease caused by *A. rolfsii*, with a lower percentage of disease incidence (19.7%) than for non-amended soil (40.7%) in vegetable soybean seedlings [21]. Soil amended with 10%, 25%, and 50% (*v*/*v*) Tiger worm vermicompost exhibited reduced chickpea seedling mortality following the infection of *Sclerotium rolfsii* [13]. Suppression activity against tomato wilt caused by *Fusarium oxysporum* f. sp. *lycopersici* has been reported when growing tomatoes in soil mixed with 30% Tiger worm vermicompost [22], as well as the inhibition of damping-off disease, either caused by *Phytophthora nicotianae* on tomato [23] or *Rhizoctonia solani* on cucumber [24]. However, there have been no reports on the use of vermicompost from either the African nightcrawler or Tiger worm for soil amendment to promote growth and yield as well as damping-off suppression on vegetable soybeans.

Vegetable soybean [*Glycine max* (L.) Merrill] is a popular leguminous crop for fresh pod consumption, especially in East and Southeast Asia [25]. It has been shown to have high nutritional value with a unique taste and fragrance [25,26]. In Thailand, the Thai vegetable soybean variety “Chiang Mai 84–2” has been improved with a pandan-like fragrance and has become of economic importance [25]. However, damping-off disease, caused by several fungal pathogens, determined significant constraints in vegetable soybean production [25]. The fungus *Athelia rolfsii* (Curzi) C. C. Tu & Kimbr. (Anamorph: *Sclerotium rolfsii* Sacc.) is an important soil-borne pathogen that causes damping-off and sclerotium rot disease in Thai vegetable soybeans [21]. It produces sclerotia that encourage survival in the soil [27] and resistance to harsh environmental conditions [28]. For this reason, pathogen persistence limits the effectiveness of disease control [29], even though combined methods have been implemented [30]. Soil amendment with vermicompost is an alternative biological disease control method, especially for organic farming systems [4].

The objective of this study is to compare the potential of two vermicompost types, derived from the African nightcrawler (*Eudrilus eugeniae* Kinberg, 1867) and Tiger worm (*Eisenia fetida* Savigny, 1826), for soil amendment to suppress damping-off disease both in vitro and in vivo. Moreover, the research aims to assess the impact of both vermicompost types on the growth and yield-related traits of the potted Thai vegetable soybean [*Glycine max* (L.) Merrill] variety “Chiang Mai 84–2” when grown in greenhouse conditions. In this study, vermicompost was added to the soil at varying application rates (1%, 2%, and 3% *w*/*w*). The AF vermicompost exhibited significantly superior suppression of damping-off disease compared to the TG vermicompost in both in vitro and greenhouse conditions. According to the results, the AF vermicompost was significantly better at suppressing both the mycelial growth of *A. rolfsii* and damping-off incidence than the TG vermicompost. The application of vermicompost at a 2% *w*/*w* through soil amendment was deemed to be appropriate for promoting the growth and yield of potted vegetable soybeans. These results offer guidance on the use of vermicompost to control soil-borne plant diseases, enhance plant growth, and improve yields, particularly in organic crop production.

## 2. Results and Discussion

### 2.1. Experiment 1: Antagonistic Activity of Vermicompost against A. rolfsii In Vitro

**Figure 1:**
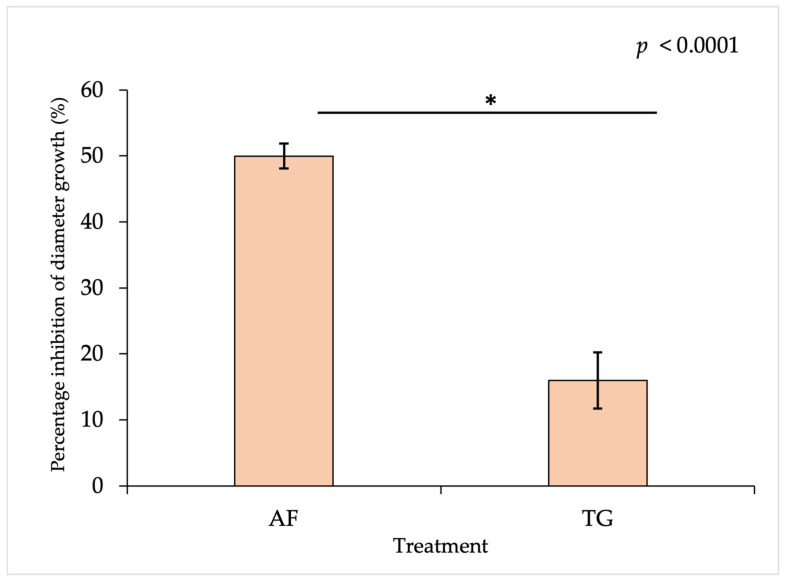
Percentage inhibition of diameter growth (PIDG) in *Athelia rolfsii,* measured on day three after culture from a modified swab plate technique using vermicompost filtrate as the biocontrol agent (mean ± standard deviation). The asterisk indicates a significant difference between treatments according to a two-sample *t*-test (*n* = 6). AF = African nightcrawler vermicompost filtrate; TG = Tiger worm vermicompost filtrate.

**Figure 2:**
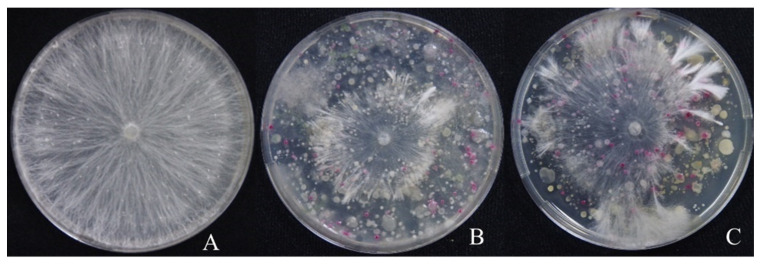
Colony of *Athelia rolfsii* was observed on day three after culture. *A. rolfsii* grown on (**A**) PDA; (**B**) African nightcrawler vermicompost filtrate over PDA; (**C**) Tiger worm vermicompost filtrate over PDA. The African nightcrawler and Tiger worm vermicompost filtrate were used at 1000-fold dilutions.

There was a significant difference in the antagonistic activities of vermicompost from the African nightcrawler (AF) and Tiger worm (TG) against the mycelial growth of *A. rolfsii* in vitro (Figure 1). Vermicompost from AF exhibited significantly higher antagonistic activity against *A. rolfsii* in vitro than vermicompost from TG, with a 50% and 16% inhibition of diameter growth, respectively (Figure 1 and Figure 2). This finding corresponded with a previous study by Nonthapa et al. [21], who observed the inhibition activity of AF vermicompost filtrate against the mycelial growth of *A. rolfsii* in vitro. The suppressive effect of vermicompost from TG on the mycelial growth of *Fusarium oxysporum* f. sp. *radicis*-*cucumerinum* and *Rhizoctonia solani* in vitro was reported using vermicompost tea [31].

Earthworm species determine the associated microbial community of respective vermicompost, indicating species-specific earthworm effects on the microbial community [32,33]. Likewise, the number of antagonistic microbial species may be diverse among vermicompost from different earthworms [34], leading to the different antagonistic activity against *A. rolfsii* in vitro shown in this study (Figure 1 and Figure 2). This suggests that the suppressiveness of vermicompost on soil-borne plant pathogens is dependent upon the earthworm species and their associated microorganisms.

Both the chemical and biotic factors of vermicompost may play a major role in suppressing activity on the mycelial growth of the fungal pathogens in vitro. However, according to the empirical studies conducted by Szczech [22], biotic factors, including antagonistic bacteria and fungi living in vermicompost, are the key players in the suppressiveness of the vermicompost against mycelial growth in vitro, rather than the impact of chemical properties. When comparing the antagonistic activity of sterile vermicompost extract to the non-sterile one, the sterile vermicompost extract lost the ability to inhibit the mycelial growth of *Fusarium oxysporum* f. sp. *lycopersici* [22]. This study isolated antagonistic *Trichoderma* sp. and *Bacillus* spp. from vermicompost (Table 1). *Trichoderma* sp. was found only in AF vermicompost, which showed a 50.8% inhibition of radial growth (PIRG). The *Bacillus* spp. isolated from AF or TG vermicompost exhibited antagonistic activity against *A. rolfsii* in vitro. Interestingly, antagonistic *Bacillus* spp. isolated from AF vermicompost revealed significantly greater PIRG than TG vermicompost (Table 1). It implied better antagonistic activity of AF vermicompost against *A. rolfsii* than TG vermicompost. Our previous experiment found antagonistic *Bacillus* spp. and *Trichoderma* sp. in vermicompost from AF [21].

### 2.2. Experiment 2: Vermicompost as a Soil Amendment for the Suppression of Damping-Off Disease

**Figure 3:**
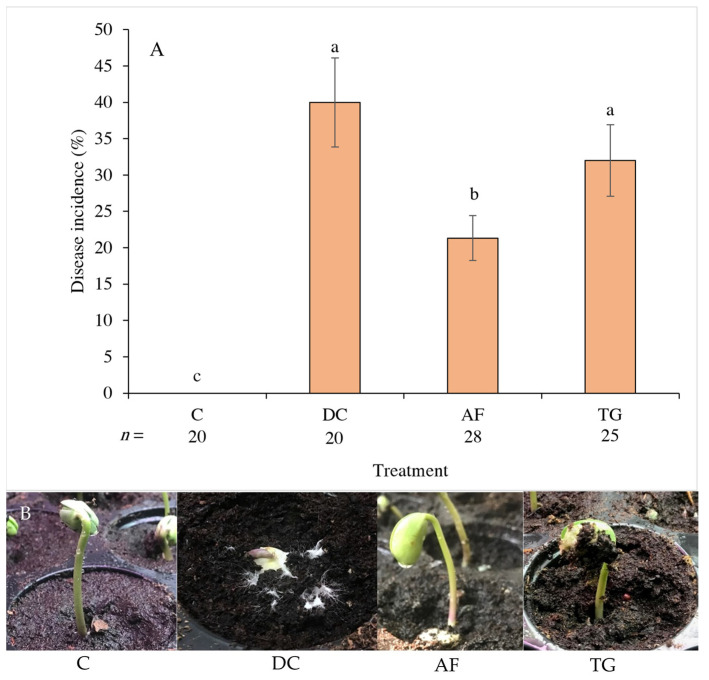
Damping-off disease incidence on soybean seedlings after inoculation with *Athelia rolfsii* under greenhouse conditions. (**A**) Percentage of disease incidence observed on soybean seedlings at day 15 after inoculation (mean ± standard deviation); (**B**) Representative soybean seedlings from each treatment were observed on day ten after inoculation. Different lowercase letters above the bars indicate significant differences between treatments according to the least significant difference (LSD) test following ANOVA (*p* < 0.05), where *n* = total number of germinated seeds of each treatment; C = non-pathogen-inoculated control; DC = pathogen-inoculated control; AF = *A. rolfsii* + African nightcrawler vermicompost; TG = *A. rolfsii* + Tiger worm vermicompost.

No post-emergence damping-off disease incidence was observed in the non-pathogen-inoculated control, but severe symptoms were found in the pathogen-inoculated control (Figure 3A,B). There was a significant difference between AF and TG vermicompost in the suppression of damping-off disease caused by *A. rolfsii* under greenhouse conditions (Figure 3A). The percentage of disease incidence observed on seedlings grown in soil amended with AF vermicompost (21.0%) was significantly lower than that for the pathogen-inoculated control (40.0%) (Figure 3A). However, soil amended with TG vermicompost provided 32% plant disease incidence, which did not differ significantly from the pathogen-inoculated control (Figure 3A). The results indicated that AF vermicompost as a soil amendment led to the lowest damping-off disease incidence on vegetable soybean seedlings among all inoculated treatments under this experimental condition. The efficacious suppression of damping-off caused by *A. rolfsii* on vegetable soybean seedlings using the AF vermicompost amendment was shown by Nonthapa et al. [21]. In this study, soil amendment with Tiger worm did not significantly reduce the incidence of damping-off disease. However, in other experiments, soil amendment with TG vermicompost, derived from various substrates, demonstrated the inhibition of damping-off disease caused by *Phytophthora nicotianae* on tomato [23], *Rhizoctonia solani* on cucumber [24], and *F. oxysporum* f. sp. *lycopersici* on Psyllium [35].

The results of testing AF and TG vermicompost as soil amendments for controlling damping-off disease under greenhouse conditions (Figure 3A) corresponded to the antagonistic activity of both vermicompost types in vitro, as shown in experiment 1 (Figure 1). In the present study, AF vermicompost showed higher efficacy in suppressing damping-off disease than TG vermicompost. In soil-borne plant disease control via soil amendment with vermicompost, antagonistic microbes provided direct suppression in the mycelial growth of fungal pathogens [19,22], relying on interference competition for resources between plant pathogens and antagonistic microbes in vermicompost [36]. Antagonistic microbes found in AF vermicompost, such as *Bacillus* spp., *Pseudomonas* spp., and *Streptomyces* spp., were more diverse than those in TG vermicompost [37]. Therefore, AF vermicompost was found to exhibit better suppressive ability than TG vermicompost, as presented in this study (Figure 1, Figure 2 and Figure 3).

Two earthworm species were fed the same materials for vermicomposting, crucially influencing the respective vermicompost’s physico-chemical properties [7] and biological properties [33]. In this study, AF vermicompost contained higher nutrient content and organic carbon than TG vermicompost (Table 2), corresponding to the report by Pattnaik and Reddy [7]. It implied that amending AF vermicompost to the soil might facilitate the exploitative competition of beneficial microbes in comparison to TG vermicompost. In addition to nutrient content, Pattnaik and Reddy [37] revealed that AF vermicompost had significantly more species diversity in microbes and biomass, especially actinomycetes, than TG vermicompost. Applying AF vermicompost rather than TG vermicompost via soil amendment might introduce a higher number of beneficial microbes. Consequently, there were limited resources for the pathogens, resulting in reduced growth and disease incidence, as revealed in the current study (Figure 1, Figure 2 and Figure 3).

On the other hand, using vermicompost as a soil amendment has the indirect effect of suppressing soil-borne plant disease. It leads to the modification of physical soil properties, affecting the multiplication of plant pathogens and the growth of beneficial microbes [19,38]. The experimental study demonstrated the depletion of *F. oxysporum* f. sp. *lycopersici* abundance with the increasing relative abundance of beneficial bacteria after long-term vermicompost application as a soil amendment [38]. Moreover, soil amended with vermicompost exhibited increased pH, NH_4_^+^-N, organic matter, dissolved organic carbon [38], and humic acid [17], positively correlating with the abundance of beneficial bacteria. These exploitative bacteria compete for resources over plant pathogens [36].

### 2.3. Experiment 4: Vermicompost as a Soil Amendment for Plant Growth and Yield Promotion

#### 2.3.1. Vermicompost as a Soil Amendment for the Promotion of Some Growth Traits

**Figure 4:**
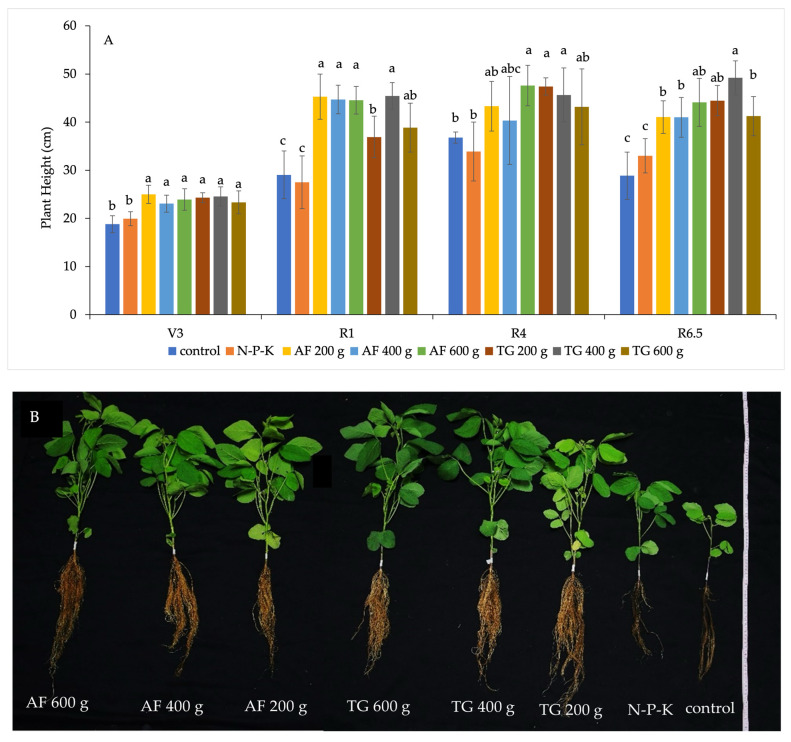

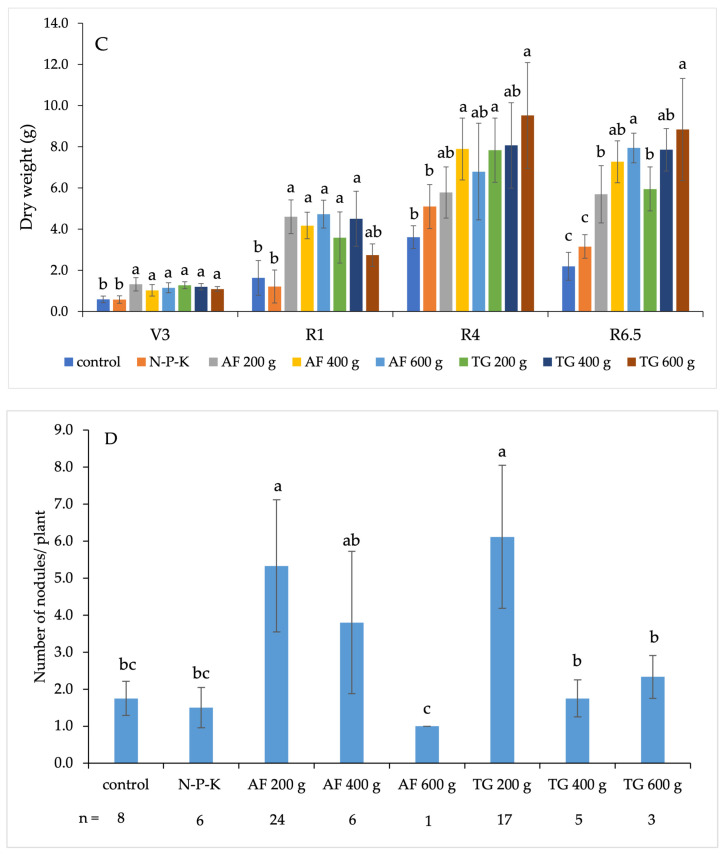
Potted vegetable soybean plant growth in soil amended with varied vermicompost rates. (**A**) Plant height and (**B**) Plant dry weight measured at four growth stages (mean ± standard deviation). (**C**) Whole plants were observed at the R3 growth stage. (**D**) Number of nodules per plant observed at the R6.5 growth stage, where *n* = number of plants producing root nodules. Different lowercase letters above the bars indicate significant differences between treatments according to Tukey’s HSD (honestly significant difference) test following ANOVA (*p* < 0.05). V3 = vegetative stage 3 (three trifoliates fully unfolded at 20 days after germination); R1 = reproductive stage 1 (starting to bloom at 31 days after germination); R4 = reproductive stage 4 (full pod at 44 days after germination); R6.5 = reproductive stage 6.5 (full seed before maturity at 76 days after germination). AF = African nightcrawler vermicompost, and TG = Tiger worm vermicompost. Ten plants per treatment were examined for plant height and dry weight at each growth stage during the experiment.

Potted vegetable soybean plants grown in soil amended with vermicompost, either produced from AF or TG, generally achieved significantly greater plant height and dry weight than control and chemical fertilizer treatment at every sampling point, relying on four growth stages (Figure 4A,B). For instance, the whole plants observed at the R3 growth stage are shown in Figure 4C. For each vermicompost type, plant heights, and dry weights at individual growth stages did not differ among treatments, varying in the quantity of vermicompost used for soil amendment (Figure 4A,B). The results suggest that adding AF or TG vermicompost at a rate of 200 g per pot is the minimal quantity needed to promote potted vegetable soybean growth under this experimental condition. The appropriate amount or rate of vermicompost for soil amendment depends on the plant species and the physico-chemical properties of the soil [4,13]. The present study demonstrated an appropriate rate of AF vermicompost and TG vermicompost for potted vegetable soybeans, precisely the Chiang Mai 84–2 variety. Soil amended with AF vermicompost was found to promote potted soybean growth at 148 kg N ha^−1^ [14]. The rates of AF and TG vermicompost application in soil amendment were adjusted for suitability based on the specific requirements of plants, such as tomato and eggplant [16]. Interestingly, the effects of AF or TG vermicompost on plant growth did not differ significantly for each rate (Figure 4A–C). The results suggest that both types of vermicompost can provide the minimum nutrient requirements (Table 2) for the plant height and dry weight of potted vegetable soybeans under these experimental conditions.

Moreover, the number of root nodules was observed in 50 plants from each treatment at the R6.5 developmental stage. The number of plants that produced root nodules varied among treatments. Plants grown in potting soil amended with AF vermicompost at the rate of 200 g per pot produced the most root nodules (24 plants), followed by those treated with TG vermicompost amended at the rate of 200 g per pot (17 plants) (Figure 4D). These two treatments demonstrated the highest number of nodules per plant (Figure 4D). The results suggest that vermicompost, at the lowest level of soil amendment in this study, can enhance root nodule formation. Maji et al. [17] also reported that mixing TG vermicompost with 9 g/kg^−1^ of soil boosted root nodulation in potted peas. Previous experiments have revealed that amending the soil with vermicompost could enrich humic acid, which has been reported to improve nodulation in soybeans [17,39]. Interestingly, increasing the rate of vermicompost amendment in soil led to fewer plants with root nodules (Figure 4D), aligning with a decrease in soybean nodulation when a higher humic acid concentration is applied [39]. AF and TG vermicompost have been found to contain a high content of organic matter, which is the source of humic acid (Table 2), suggesting that a concentration of vermicompost at 200 g per pot would be preferable for plant growth promotion [4]. Our findings have important implications for agricultural practices in optimizing the use of vermicompost for better plant growth and root nodulation.

Furthermore, the exploration of bacterial communities in vermicompost revealed the presence of several species of nitrogen-fixing bacteria, varying according to the earthworm species [3,19]. The other AF and TG vermicompost sources presented symbiotic nitrogen-fixing bacteria, *Acinetobacter* spp., *Enterobacter* spp., and *Klebsiella* spp., in greater abundance than in the case of AF vermicompost [37]. Moreover, several non-symbiotic nitrogen-fixing bacteria, such as *Azotobacter* and *Clostridium*, were detected in the AF and TG casts [40]. This study demonstrates that vegetable soybeans treated with AF vermicompost produce more root nodules than those treated with TG vermicompost (Figure 4D). 

#### 2.3.2. Vermicompost as a Soil Amendment for Yield Promotion

**Figure 5:**
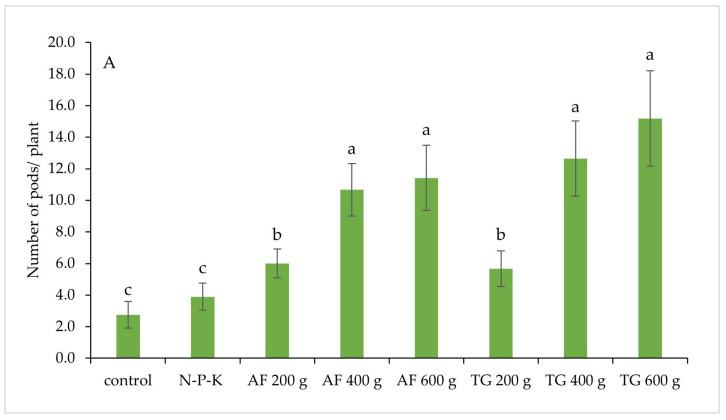

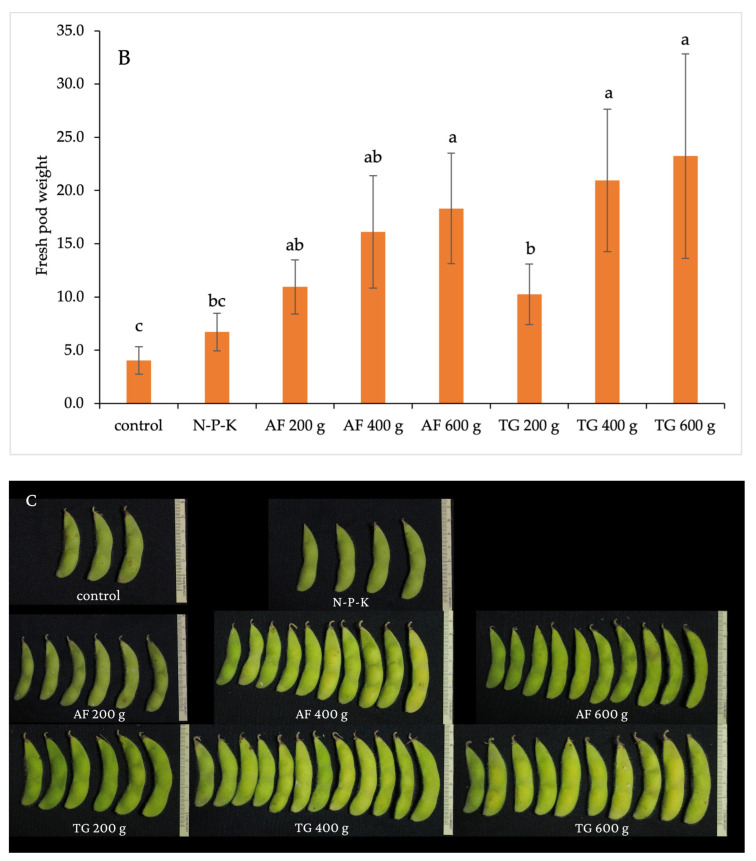
Yields of potted vegetable soybean plants grown in soil amended with varying vermicompost ratios. (**A**) Pods per plant and (**B**) Fresh pod weight per plant measured at the R6.5 growth stage (mean ± standard deviation). (**C**) Fresh pods in each treatment were observed at the R4 growth stage. Different lowercase letters above the bars indicate significant differences between treatments according to Tukey’s HSD (honestly significant difference) test following ANOVA (*p* < 0.05). AF = African nightcrawler vermicompost, and TG = Tiger worm vermicompost.

Potted soybean plants grown in soil amended with each rate of AF or TG vermicompost showed a significantly higher number of pods per plant and total fresh pod weight per plant than those subject to control and chemical fertilizer treatment (N–P–K) (Figure 5A–C). This indicated that adding the vermicompost at a rate of 200 g per pot was the minimal requirement for yield promotion compared to the control under this experimental condition. In each vermicompost type, plants grown in soil amended with 400 g and 600 g vermicompost per pot produced significantly greater yields (number of pods per plant and total pod fresh weight per plant) than those amended with 200 g vermicompost (Figure 5A,B). Measured plant yields did not differ between the two AF and TG vermicompost treatments (Figure 5–C). This suggested that AF and TG vermicompost at a rate of 400 g per pot was a sufficient rate for promoting the yield of potted vegetable soybean. At every rate of vermicompost application, there was no difference in plant yields between treatments using AF and TG vermicompost (Figure 5A,B).

The use of AF vermicompost as a potting media amendment has been revealed to effectively promote the yields of potted soybeans [14], tomatoes, and eggplants [14,15,16]. Likewise, TG vermicompost as a potting media amendment has been found to promote the yields of peas [17], tomatoes, and eggplants [16]. Since vermicompost has a high content of nutrients, organic matter [41], and humic acid [17], its use in soil amendment can improve soil fertility and soil nutrients, mainly nitrogen, phosphorus, and potassium [41]. The vermicompost used in this study contained more nutrients and organic matter than pure soil, as shown in Table 2. Moreover, vermicompost led to a change in pH, enhancing nutrient availability, especially phosphorus [42]. Therefore, vermicompost benefits the growth and yield of plant species and the physico-chemical properties of soil [4,13]. An excess concentration of vermicompost could cause high electrical conductivity, which might negatively affect plant performance [4]. The present study reveals that the rate of AF and TG vermicompost at 400 g per pot is the most suitable for promoting vegetable soybean yield.

The biological factors, such as specific microbial communities in vermicompost, benefit plant growth through the different functional traits of specific microbial groups [19]. For example, nitrogen-fixing bacteria promote plant growth by facilitating nitrification and phosphate solubilization [12,40]. 

## 3. Materials and Methods

### 3.1. Plant Material

Seeds of the Thai vegetable soybean [*Glycine max* (L.) Merrill variety “Chiang Mai 84–2”] were obtained from the Department of Agronomy, Faculty of Agriculture, Khon Kaen University, Thailand.

### 3.2. Soil

The soil used in the pot trials under greenhouse experiments came from the Ban Phai series, characterized as pale brown, siliceous, loamy sand [43]. It was collected from an agricultural field located in Ban Had District, Khon Kaen Province, Thailand. The basic physico-chemical properties of the soil were analyzed as described by Nonthapa et al. [21] and presented in Table 2.

### 3.3. Vermicompost

Two vermicompost types were used in the present study, the African nightcrawler (AF) and Tiger worm (TG), derived from the African nightcrawler (*Eudrilus eugeniae* Kinberg, 1867) and Tiger worm (*Eisenia fetida* Savigny, 1826), respectively. The prepared AF and TG vermicomposts were provided by the Research, Developing, and Learning Center on Earthworms for Agriculture and Environment, Integrated Water Resource Management Research and Development Center at Khon Kaen University in Northeast Thailand. The vermicompost was produced according to Iwai et al. [44], while the vermicomposting materials for both AF and TG vermicompost included cassava peel and cow manure for each earthworm species. Both vermicompost types used in this investigation were collected after 45 days of composting and obtained from the same batch. The AF vermicompost contained higher physico-chemical properties, i.e., EC, organic matter, organic carbon, C/N, nitrogen, total P, and total K, than the TG vermicompost (Table 2).

### 3.4. Fungal Pathogen Isolation and Inoculum Preparation for the Greenhouse Experiment

The phytopathogenic fungus *Athelia rolfsii* was isolated from infected vegetable soybean plants [*Glycine max* (L.) Merrill variety “Chiang Mai 84–2”] collected from the agricultural fields at Khon Kaen University, Khon Kaen Province, Thailand. The diseased plants were incubated at 25 ± 2 °C (KBWF720, BINDER, Tuttlingen, Germany) and 90% hygrometry in a moist chamber to induce sclerotium production [45]. Direct sclerotium isolation was employed following the method described by Nonthapa et al. [21]. After surface sterilization with 5% sodium hypochlorite for one minute, sclerotia were rinsed two to three times in distilled water. They were then transferred onto a potato dextrose agar medium (PDA; HiMedia; Mumbai, India) in a 90 mm diameter Petri dish (Hycon Sterilized Petri Dish Plastic 90 × 15 mm, Biomed Co., Ltd., Bangkok, Thailand) and incubated at 25 ± 2 °C in the incubator (KBWF720, BINDER, Tuttlingen, Germany) for five days [46]. The pure culture of *A. rolfsii* was maintained on a PDA slant. The fungal pathogen inoculum for greenhouse experiments was prepared according to the method depicted by Sennoi et al. [47]. The PDA plug (5 mm in diameter) with growing *A. rolfsii* mycelia was transferred to 30 g of sterilized sorghum seeds in an Erlenmeyer flask (250 mL Erlenmeyer flask PYREX^TM^, Glendale, AZ, USA) and incubated at 25 ± 2 °C (KBWF720, BINDER, Tuttlingen, Germany) for two weeks [47].

### 3.5. Experiment 1: In Vitro Fungal Confrontation Assays Using Vermicompost Filtrate

The AF and TG vermicomposts were placed into the vermicompost filtrate through Whatman^®^ grade 1 qualitative filter paper (Whatman, Cytiva, Amersham, UK) [48] before being used in the confrontation assay against the mycelial growth of *A. rolfsii.* Following the method described in Nonthapa et al. [21], a modified swab plate technique was used to test the antagonistic activity of AF or TG vermicompost against the mycelial growth of *A. rolfsii*. The filtrate of each vermicompost type was spread on a PDA (HiMedia; Mumbai, India) prior to placing the agar plug of *A. rolfsii*. The Petri dishes (Hycon Sterilized Petri Dish Plastic 90 × 15 mm, Biomed Co., Ltd., Bangkok, Thailand) were incubated at 25 ± 2 °C (KBWF720, BINDER, Tuttlingen, Germany) and observed daily at 4 p.m. for diameter growth of the fungal pathogen until day three after culture. The experiment was arranged in a completely randomized design (CRD) with three replications. The percentage inhibition of diameter growth (PIDG) was calculated following the formula PIDG = [(D_1_ − D_2_)/D_1_] × 100, where D_1_ is the diameter growth of *A. rolfsii* on the control Petri dish (pure PDA), and D_2_ is the diameter growth of the fungal pathogen on the Petri dish spread with vermicompost filtrate [49].

### 3.6. Experiment 2: Antagonistic Activity of Isolated Microbes against A. rolfsii

Vermicompost was placed into the filtrate as described by Gopalakrishnan et al. [48]. Bacteria were isolated and multiplied on nutrient agar (NA) (HiMedia; Mumbai, India) at 25 ± 2 °C for 48 h, and fungi were isolated and cultured on PDA (HiMedia; Mumbai, India) at 25 ± 2 °C for seven days. A dual culture assay between agar plugs of *A. rolfsii* and individual isolated microbes was conducted on a PDA (HiMedia; Mumbai, India) by placing each plug 2 cm from the border on opposite sides of a 9 cm diameter Petri dish (Hycon Sterilized Petri Dish Plastic 90 × 15 mm, Biomed Co., Ltd., Bangkok, Thailand) [50]. Agar plugs of *A. rolfsii* were prepared as described in *3.4*. To test the antagonistic activity, agar plugs of isolated fungi were used in a dual culture assay [50]. For antagonistic bacteria, the bacterial colony was established in a dual culture assay by the streak method [51]. The plates were incubated at 25 ± 2 °C (KBWF720, BINDER, Tuttlingen, Germany) and observed daily for radial growth of *A. rolfsii*. The percentage inhibition of radial growth (PIRG) was calculated following Muniroh et al. [52]: PIRG = [(R_1_ − R_2_)/R_1_] × 100, with R_1_ being the radial growth of *A. rolfsii* on the control plate and R_2_ the radial growth of the fungal pathogen on the dual culture Petri dish. The experiment was assigned to a CRD with five replications.

### 3.7. Experiment 3: Suppression of Damping-Off Disease under Greenhouse Conditions

The peat moss Potgrond H^®^ (Klasmann–Deilmann, Geeste, Germany) was the potting medium used in the biocontrol assay. Each pot (4 cm in height and 5 cm in diameter) contained 20 g of potting media. For treatments with vermicompost, the peat moss was amended with 20% (*v*/*v*) of vermicompost before pot filling, following Aggeli et al. [53]. One grain of sorghum colonized by the mycelia of *A. rolfsii* was used as the fungal pathogen inoculum for each pot [21]. The inoculation of *A. rolfsii* on vegetable soybeans was carried out simultaneously with single seed sowing in each plastic pot [46]. All pots were kept in a growth chamber at 25 ± 2 °C average temperature and 90% relative humidity [54], with a light intensity of 1412.8 cd (12/12 h day/night) [21]. The experiment was carried out 21 days after inoculation. Disease incidence on vegetable soybean plants was observed and recorded at least once each day until day 15 after inoculation. Four treatments were evaluated during the experiment: (1) a non-pathogen-inoculated control; (2) a pathogen-inoculated control (only *A. rolfsii*); (3) *A. rolfsii* + AF vermicompost; and (4) *A. rolfsii* + TG vermicompost. Disease incidence (DI) was calculated following the previous study of Pandey et al. [55]. The treatment was performed in a CRD with five replications. For each replication, seven pots containing one plant were observed for disease incidence.

### 3.8. Experiment 4: Vermicompost as a Soil Amendment for Plant Growth and Yield Promotion

Two seeds of vegetable soybean were sown in each plastic pot (16.51 cm in height and 22.86 cm in diameter) containing 20 kg of soil or soil amended with vermicompost. Either AF or TG vermicompost was used as a soil amendment. For each type of vermicompost, three rates of substitution, including 200 g (1% *w*/*w*), 400 g (2% *w*/*w*), and 600 g (3% *w*/*w*) per pot, were employed in different treatments. There were eight treatments, as follows: (1) 20 kg pure soil, (2) 20 kg pure soil + N–P–K chemical fertilizer (the chemical fertilizer was applied at 14.06 kg/ha (N_2_–P_2_O_5_–K_2_O) 15 days after germination [56], (3) AF 200 g substitution per pot, (4) AF 400 g substitution per pot, (5) AF 600 g substitution per pot, (6) TG 200 g substitution per pot, (7) TG 400 g substitution per pot, (8) TG 600 g substitution per pot. The experimental design was assigned to a CRD. Each treatment was performed on thirty pots, each containing two plants.

The vegetable soybean plants were kept in a greenhouse under field conditions at the agronomy field crop station, Faculty of Agriculture, Khon Kaen University, Khon Kaen, Thailand (16°28′27.7″ N, 102°48′36.5″ E; 190 m above sea level) during the rainy season (June–August 2021). Plant height, plant dry weight, and the number of nodules were measured at vegetative stage 3 (V3: three trifoliates fully unfolded at 20 days after germination), reproductive stage 1 (R1: starting to bloom at 31 days after germination), reproductive stage 4 (R4: full pod at 44 days after germination), and reproductive stage 6.5 (R6.5: full seed before maturity at 76 days after germination). Five replications of each treatment were collected for trait measurement at each growth stage and then removed from the experiment. The vegetable soybean yields were measured at the R6.5 growth stage according to the number of pods per plant and fresh pod weight per plant.

### 3.9. Statistical Analysis

Data were expressed as mean ± standard deviation (SD). The statistical analyses were conducted using R Studio Desktop 4.2.1 and the R software package version 4.3.3 [57]. A two-sample *t*-test was performed using the package “dplyr” version 1.1.4 [58] to determine any significant difference between the PIDGs of the two treatments, namely the pathogen’s responses to AF and TG vermicompost (Experiment 1). The data on PIRGs from different isolated microbes showing antagonistic activity against *A. rolfsii* were analyzed using one-way analysis of variance (ANOVA), followed by the least significant difference (LSD) test at a 95% significance level (Experiment 2). For the inoculation experiment (Experiment 3), the DI data from different treatments were subjected to an ANOVA, followed by an LSD test at a 95% significance level. The plant growth and yield data from different treatments were performed through ANOVA and subsequently analyzed via Tukey’s HSD (honestly significant difference) test at the 95% significance level (Experiment 4). All one-way ANOVA and pairwise comparisons were performed using the package “agricolae” version 1.3–5 [59]. 

## 4. Conclusions

This study revealed a significant breakthrough in the understanding of vermicompost, showing its dual purpose as a soil amendment. The AF vermicompost performed better than the TG vermicompost in inhibiting *A. rolfsii* mycelial growth in vitro and suppressing damping-off disease under greenhouse conditions. Vermicompost as a soil amendment at 2% (*w*/*w*) was found to be suitable for promoting the growth and yield of potted vegetable soybeans under this experimental condition for AF and TG vermicompost. Based on the dual purpose of vermicompost utilization, the AF vermicompost was preferred for suppressing damping-off disease and promoting potted vegetable soybeans. The increase in vermicompost utilization will encourage the conversion of organic waste to crop nutrients in accordance with eco-friendly, sustainable agriculture.

## Figures and Tables

**Table 1:** Percentage inhibition of radial growth (PIRG) measured from a dual culture assay between antagonistic microbes isolated from two vermicompost types and *A. rolfsii* at day five after culture on potato dextrose agar with five replications (mean ± standard deviation).

Vermicompost	Isolated Microbes	PIRG (%)
African nightcrawler (AF)	*Trichoderma* sp. isolate AF1	50.8 ± 3.4 ^a^
	*Bacillus* sp. isolate AF1	43.5 ± 1.0 ^b^
	*Bacillus* sp. isolate AF2	41.3 ± 6.1 ^b^
Tiger worm (TG)	*Bacillus* sp. isolate TG3	24.5 ± 1.0 ^c^
	*Bacillus* sp. isolate TG4	16.9 ± 3.9 ^d^

Means with different superscript letters indicate significant differences between the tested antagonistic microbes according to the least significant difference (LSD) test following ANOVA (*p* < 0.05).

**Table 2:** The basic physico-chemical properties of soil and vermicompost used in the pot trial experiments with three replications (mean ± standard deviation).

Parameter	Soil	AF	TG
pH (1:1)	6.70 ± 0.01	-	-
pH (1:10)	-	6.61 ± 0.01	6.74 ± 0.00
EC (dS/cm)	0.05 ± 0.00	4.05 ± 0.01	3.29 ± 0.00
OM (%)	0.18 ± 0.01	28.24 ± 0.16	13.27 ± 0.35
N (%)	0.01 ± 0.00	1.33 ± 0.04	1.15 ± 0.05
Available P (mg/kg)	25.75 ± 0.35	-	-
Exchangeable K (mg/kg)	21.72 ± 1.74	-	-
Total P (g/kg)	-	47.00 ± 0.00	40.00 ± 0.00
Total K (g/kg)	-	41.00 ± 0.01	38.00 ± 0.00
OC (%)	0.11 ± 0.01	16.37 ± 0.09	7.69 ± 0.20
C/N	15.3 ± 0.98	12.32 ± 0.49	6.72 ± 0. 46

AF = vermicompost from the African nightcrawler, and TG = vermicompost from the Tiger worm. EC = electrical conductivity; OC = organic carbon; C = carbon; N = nitrogen; K = exchangeable potassium; P = available phosphorus; exchangeable potassium and available phosphorus, measured for soil; total potassium and total phosphorus, measured for vermicompost.

## Data Availability

The datasets obtained and analyzed in the current study are available from the corresponding author upon reasonable request.
